# Copper-Contaminated Substrate Biosorption by *Penicillium* sp. Isolated from Kefir Grains

**DOI:** 10.3390/microorganisms11061439

**Published:** 2023-05-30

**Authors:** Antonio Ferreira de Oliveira, Raquellyne Baia Machado, Adriana Maciel Ferreira, Iracirema da Silva Sena, Maria Eduarda Silveira, Ana Maria Santos de Almeida, Francinaldo S. Braga, Alex Bruno Lobato Rodrigues, Roberto Messias Bezerra, Irlon Maciel Ferreira, Alexandro Cezar Florentino

**Affiliations:** 1 Ichthyo and Genotoxicity Laboratory, Department of Exact and Technological Sciences, Federal University of Amapá, Rod. JK, km 02, Macapá 68903-419, Brazil; antonoliver2607@yahoo.com (A.F.d.O.); raquellyne.machado@gmail.com (R.B.M.); maddu.silveira08@gmail.com (M.E.S.); anamariasantospelaes@gmail.com (A.M.S.d.A.); fsbraga@unifap.br (F.S.B.); messias@unifap.br (R.M.B.); 2 Research Laboratory of Drugs, Department of Biological and Health Sciences, Federal University of Amapá, Rod. JK, km 02, Macapá 68903-419, Brazil; adriana.maciel@gmail.com; 3 Laboratory of Biocatalysis and Applied Organic Synthesis, Department of Exact and Technological Sciences, Federal University of Amapá, Rod. JK, km 02, Macapá 68903-419, Brazil; ciremasena@gmail.com (I.d.S.S.); irlon.ferreira@gmail.com (I.M.F.); 4 Analytical Chemistry Laboratory, Department of Exact and Technological Sciences, Federal University of Amapá, Rod. JK, km 02, Macapá 68903-419, Brazil; alex.rodrigues@unifap.br

**Keywords:** bioremediation, copper nitrate, *Penicillium* sp., kefir, inorganic contaminant

## Abstract

In this bioremediation study, the fungus *Penicillium* sp. isolated from kefir grains was evaluated for its resistance to copper in the culture medium. *Penicillium* sp. was cultivated in liquid medium prepared using 2% malt-agar at pH 7.0. Biomass of the fungus was significantly reduced, but only when 800 mg·L^−1^ of Cu(NO_3_)_2_ copper nitrate was used. The effect on radial growth of the fungus in experiments combining different pH values and the inorganic contaminant showed an inhibition of 73% at pH 4.0, 75% at pH 7.0 and 77% at pH 9.0 in liquid medium. Thus, even though the growth of *Penicillium* sp. could be inhibited with relatively high doses of copper nitrate, images obtained with scanning electron microscopy showed the preservation of fungal cell integrity. Therefore, it can be concluded that *Penicillium* sp. isolated from kefir grains can survive while performing bioremediation to minimize the negative effects of copper on the environment through biosorption.

## 1. Introduction

The continuous efflux of heavy metals from industry pollutes the environment and threatens public health [1]. Anthropogenic activities, such as spraying cupric fungicides [2], prospecting and mining, have saturated soil, water and sediment levels with copper (Cu), often with a high degree of toxicity [3,4]. High concentrations of copper and other heavy metals in industrial wastewater have caused many serious environmental and health problems [5,6].

Copper is one of the essential elements for all living organisms, including humans. It acts as a cofactor for many metalloenzymes involved in hemoglobin formation and carbohydrate metabolism [7]. However, copper can contaminate large areas, mainly through mining wastewater [8], and drinking contaminated water can be life-threatening.

In a recent work, Kamal et al. [9] highlight the use of fungi as an important strategy against inorganic contaminants. They also emphasize that different species of fungi make up a powerful arsenal with the potential for use in bioremediation. More specifically, fungal mycelium has an extensive and high reactive surface area in contact with the environment. These characteristics afford fungi the potential for both biodegradation and biosorption. Furthermore, fungal physiology is characterized by a high capacity for stress, as well as the production of enzymes and secondary metabolites [10]. 

Kefir, a yogurt-like fermented milk drink made from kefir grains [11], is a microbial complex consisting of fungi with high biosynthetic capacity in symbiosis with lactobacilli with high catabolic capacity [12]. This study aimed to evaluate the copper tolerance of *Penicillium* sp. obtained from kefir grains to determine its use in bioremediation. This is an extension of our previous work that examined the consumption of kefir to protect against the toxicity of heavy metals. This step also represents the initial phase in the selection of candidate microorganisms for use in the bioremediation of copper-contaminated environments.

## 2. Materials and Methods

### 2.1. Isolation of Penicillium sp. from Kefir Grains

Kefir samples were collected from the Research Laboratory of Drugs of the Federal University of Amapá (UNIFAP). The isolated microorganism used in the study was obtained from kefir grains. To obtain the isolate, malt-agar extract (2%) with antibiotic insertion (chloraphenicol) was used. Approximately 10 g of kefir grains were ground with a vortex device (Instrucamp/Ika Vortex/Campinas, Brazil). After a two-minute pause, an inoculation loop was used to insert the kefir supernatant into Petri dishes previously filled with culture medium. Then the Petri dishes were incubated in a Biochemical Oxygen Demand (B.O.D.) chamber (Being/Bio-RWP/Shanghai, China). The experiment was maintained under conditions of a 12 h photoperiod and temperature of 26 ± 1 °C. Following a seven-day incubation, the microorganism was identified at the genus level through the analysis of colonies and morphological structures (conidiophores and conidia) based on morphological keys of sections and species developed by Gams and Bisset [13]. 

### 2.2. Culture Medium Preparation and Radial Growth Measurement

A 2% malt-agar medium at pH 7.0 was used to cultivate the fungus. Approximately 25 mL of culture medium were poured into Petri dishes (90 mm) in which *Penicillium* sp. strains were inoculated in triplicate using a bacteriological loop. The minimum inhibition concentration (MIC) was calculated to analyze the influence of copper (Cu^2+^) concentration on the microorganism’s growth.

### 2.3. Identifying Undesirable Properties of Copper Compounds through Toxicological Forecast 

The http://tox.charite.de/tox (accessed on 7 October 2022) server was used to scrutinize life-threatening properties of copper compounds. To accomplish this, functional groups were compared to find molecules of interest with similar properties. The toxicological properties of copper were also analyzed according to toxicological class, capacity to generate toxic fragments, and LD_50_ values.

### 2.4. Biomass Production of Penicillium sp. and Determination of Minimum Inhibitory Concentration (MIC)

Selected strains of *Penicillium* sp. were inoculated using a bacteriological loop on malt-agar medium incubated at room temperature (27 ± 1 °C) for the preparation of biomass. After 5 days, a small portion (0.5 mm) of fungal mass was transferred to Erlenmeyer flasks (250 mL) containing 100 mL of malt-agar medium (2%) supplemented with different concentrations of the target metal (400, 600, 800, and 1000 mg·L^−1^ of Cu(NO_3_)_2_) and then incubated for 120 h in triplicate at 27 ± 1 °C. Then, the biomass was filtered through Whatman Number 1 filter paper and washed with deionized water.

Dehydration of the collected mycelium was carried out in an oven (Carbolite Gero/AX 60, Rio de Janeiro, Brazil) at 60 °C for 48 h, and the dry weight was measured using a digital scale accurate to 0.1 m. Inhibition of biomass production was calculated on a dry weight basis using the following Formula (1), where *PI* is the inhibition percentage, X is the control biomass with 0.0 ppm of the target metal, and Y is the sample biomass with the respective heavy metal.

(1) $$PI=\left(\frac{X(X-Y)}{X}\right)\times 100$$

The minimum inhibitory concentration (MIC) of copper, causing 50% growth inhibition (MIC_50_) of selected *Penicillium* sp. isolates, was calculated based on growth inhibition. Growth was measured using the halo method with a Digimess digital caliper (DIGIMESS World Tools, product no: 006797/Joinvile, Brazil). After the first 24 h, halos were formed every 12 h up to 120 h when growth of the control experiment covered the entire surface of the Petri dish. The control contained only the culture, while the target samples were placed at 400, 600, 800, and 1000 mg·L^−1^ copper Cu(NO_3_)^2^. 

### 2.5. Cultivation of Penicillium sp. in Solid Medium and Minimum Inhibitory Concentration (MIC)

To determine growth within the surface diameter of the Petri dish (90 mm), *Penicillium* sp. Was inoculated with an inoculation loop in the medium prepared with malt-agar (2%), during an incubation period of 120 h at room temperature (27 ± 1 °C). This culture medium was supplemented with different concentrations of Cu(NO_3_)_2_ (0, 200, 400, 600, 800, and 1000 mg·L^−1^). Growth was monitored by measuring the growth halo using a digital caliper. Formula (2) was used to determine growth inhibition, where MIC is the minimum inhibitory concentration and X is the radial growth medium of the control free of inorganic contaminants.

(2) $$MIC=\left(\frac{X-Y}{X}\right)\times 100$$

### 2.6. Penicillium sp. Biomass and Quantification of Copper

Malt-agar medium (2%) was prepared for cultivation of *Penicillium* sp. at room temperature (27 ± 1 °C) for 120 h. Next, an agitator was used at 75 rpm (LabFriend/VKS 75A/Barueri, Brazil) to produce biomass. Then, a 0.5 mm portion of the fungus cultivated in solid medium was inserted into several Erlenmeyer flasks (250 mL) with 100 mL of malt medium (2%), and different concentrations of copper nitrate (0, 400, 600, 800, and 1000 mg·L^−1^) were added. Incubation was performed in triplicate at a temperature of 27 ± 1 °C for 120 h.

After cultivation, filtration was performed to isolate the biomass obtained using Whatman No. 1 filter paper and washed with deionized water to remove the biomass. The mycelium obtained was placed in an oven at 60 °C for 48 h for dehydration. Dry weight was measured on a digital scale. The pH of the filtrate was also measured. To quantify remaining copper in the culture medium, an atomic absorption spectrophotometer AAS Shimadzu model 6300 (KLAB Daejeon, Republic of Korea was used. Biomass of the microorganism was cultivated for 120 h in medium agitated in a shaker at 75 rpm (LabFriend/VKS 75A/Barueri, Brazil), dehydrated, and then submitted to analysis by SEM (TM3030Plus, Hitachi, Japan).

### 2.7. Estimation of Residual Metals in the Culture Medium

After a period of 72 h, the biomass of each individually cultivated isolate was measured, respectively, in Cu(NO_3_)_2_ concentrations (0, 400, 600, 800, and 1000 mg·L^−1^). Remaining copper concentration in the culture medium was measured. 

### 2.8. Scanning Electron Microscopy (SEM) Analysis

*Penicillium* sp. mycelium isolated from kefir grains was analyzed by SEM with the aim of assessing possible differences between samples inoculated with different concentrations of Cu(NO_3_)_2_ and control samples in stirred or shaken liquid media.

### 2.9. Statistical Analysis

Experiments were carried out in a randomized factorial design in triplicate. Data are expressed as mean ± (SE). Analysis of variance (ANOVA) was performed using Tukey’s post hoc test. ANOVA assumptions of normality and homoscedasticity were tested using the Shapiro–Wilk and Bartlett tests, respectively. Analysis was performed using R 3.4.3 software (R Core Team, 2017), and *p* ≤ 0.05 was considered significant.

## 3. Results

### 3.1. Effects of Copper on the Growth of Penicillium sp.

The impact of different concentrations of copper nitrate on the growth of *Penicillium* sp. was visible by the surface diameter of culture medium in the Petri dish, and the results are summarized in Table 1. The minimum inhibitory concentration (MIC) was 43% (51.05 ± 0.50 mm) when copper concentration reached 500 mg·L^−1^, and it increased to 75% (22.17 ± 1.75 mm) at a concentration of 800 mg·L^−1^. ANOVA indicated significant differences among concentrations (F = 8.57; *p* < 0.05). Then, the Tukey test was performed using R 3.4.3 software (R Core Team, 2017). Differences were considered significant when *p* ≤ 0.05. Total inhibition of microorganismal growth was only achieved when copper nitrate reached a concentration of 1000 mg·L^−1^. These data show that this fungal microorganism is very tolerant to high doses of copper nitrate and, therefore, has the capacity to absorb copper nitrate in equally high doses. 

### 3.2. Growth in pH 4.0, 7.0, and 9.0

The effects of pH on the growth of *Penicillium* sp. were evaluated with the medium at pH 4.0, 7.0, and 9.0 (Table 2). No growth occurred at pH 2.0. However, when pH was combined with copper nitrate, radial growth of the fungus was affected with inhibition of 73% at pH 4.0, 75% at pH 7.0, and 77% at pH 9.0 relative to control.

### 3.3. Toxicological Prediction

Toxicological prediction was performed (Table 3 to compare the ability of microorganisms to tolerate the inorganic contaminant. The lethal concentration (LC_50_) of copper nitrate to mammals is 25 mg·kg^−1^ (http://tox.charite.de/tox server, accessed on 7 October 2022). Considered as Toxicological Class 2, this compound is not carcinogenic, but it is considered toxic when ingested or comes in contact with the skin. Pulmonary toxicity is probably related to the oxidative effects of copper in the lungs. Chronic exposure to copper can damage the liver and kidneys with symptoms like those found in Wilson’s disease, a genetic disorder that prevents the body from removing extra copper [14].

Using the toxicity prediction program (http://tox.charite.de/tox/ accessed on 7 october 2022), the molecular polar surface area (PSA) for Cu(NO_3_)_2_ was calculated. PSA is a very useful parameter for predicting drug transport. It also represents the sum of bridges formed by the polarity of the surface of atoms, which is related to intestinal absorption capacity and the ability to break through the blood–brain barrier (BBB).

### 3.4. Culture of Penicillium sp. in Liquid Medium

*Penicillium* sp. was inoculated in a 2% pH 7 malt-agar culture medium. A clear decline in biomass growth was observed when 800 mg·L^−1^ of copper nitrate (Cu(NO_3_)_2_) were added to liquid medium, showing that copper affected growth rate of the fungus. Decreasing pH value in the liquid medium also played a role in decreasing the microorganism’s growth rate (Table 4).

### 3.5. Scanning Electron Microscopy (SEM) Analysis of Penicillium sp. Biomass Isolated from Kefir Grains

**Figure 1:**
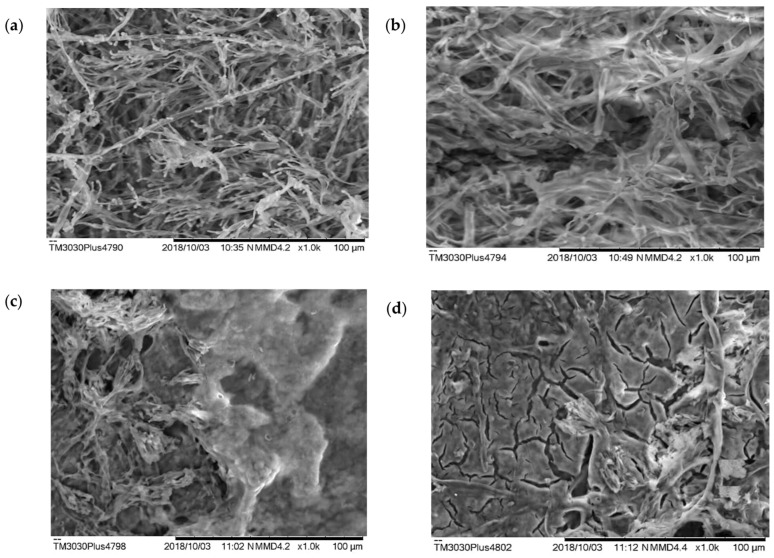
Scanning electron microscopy (SEM) analysis shows the difference in hyphal growth in *Penicillium* sp. grown in liquid medium (**a**) without copper; (**b**) with 400 mg·L^−1^; (**c**) with 600 mg·L^−1^; and (**d**) and with 800 mg·L^−1^ of Cu(NO_3_)_2_.

SEM analysis showed the changes in fungal mycelium with increasing concentrations of copper nitrate in the liquid medium (Figure 1a–d). In particular, cracks in the mycelium owing to the accumulation of heavy metal inside could be observed (Figure 1d).

## 4. Discussion

Our study shows that *Penicillium* sp. Isolated from kefir grains can bioremediate copper from contaminated environments. Heavy metal pollution is a growing problem caused by human activities [4,15]. Some heavy metals, such as copper, are essential to living organisms, while others, such as lead (Pb), cadmium (Cd), and mercury (Hg) [8,16,17,18], are nonessential and damaging to living organisms in any concentration. As noted, copper is an inorganic compound that is essential, but also nonessential. Since the ions of nonessential inorganic pollutants, including copper, have the same value as those of essential ions, the pollutants can be absorbed by the body and have damaging effects. More specifically, pollutants considered essential to basic biochemical activities in low doses, such as zinc (Zn), iron (Fe), and copper, become toxic at high concentrations [8,15,16,17,18]. 

Copper belongs to the second category of pollutants and is found in very small amounts in the human body. It plays a vital role in the production of energy in cells. Copper is a component of myelin, the lipid layer that protects neurons. It also ensures the maintenance of blood and the functioning of various enzymes in the body [19]. Copper helps produce some blood cells, hormones, and antioxidant enzymes that help synthesize neurotransmitters, form myelin sheaths, and regulate gene expression [19]. However, higher concentrations of copper have some adverse effects that can lead to Alzheimer’s disease and liver damage [20]. In this study, growth of the fungus *Penicillium* sp. Decreased when 800 mg·L^−1^ copper nitrate was used, while at 400 mg·L^−1^, the reduction in fungal growth was only 19%; growth inhibition was 75% at 800 mg·L^−1^, but 100% at 1000 mg·L^−1^. These results mean that environments overly saturated with copper need to be monitored with an eye toward the initiation of remediation programs. Indeed, several chemical methods for eliminating inorganic for pollutants have been tried, but these have carried high costs, along with the irony of causing damage to the very environment they were intended to conserve [21]. 

On the other hand, bioremediation is a low-cost biotechnological approach to avoid environmental damage [22]. Bioremediation of heavy-metal-contaminated soils using microorganisms has attracted the attention of researchers because it has been proven effective, economical, and environmentally safe [12,15]. As rightly pointed out by Sahu [23], bioremediation is the most promising technique for remediating contaminated environments.

In general, bioremediation can be performed in two ways. In one case, impurities adhere to the cell membranes of living organisms, but this results in microbial death. In another case, bioremediation occurs inside cells and results in cellular metabolism of inorganic pollutants; this is called biosorption. The term biosorption is used when the sorbent material is of biological origin, and the bioremediation process takes place in living cells [24,25] where uptake and removal involve the transport of metal ions across membrane barriers and subsequent intracellular accumulation. This is a slow and irreversible metabolic process that only occurs in living cells. Pollutants are metabolized intracellularly during uptake and usually accumulate in vacuoles [26]. 

It was recently discovered that several strains of filamentous fungi could be successfully used in bioremediation owing to their strong ability to biosorb heavy metals, especially copper and cobalt [25]. Tolerance of microorganisms to copper can be attributed to their capacity for biosorption and chelation [26]. In the present report, *Penicillium* sp. showed a high capacity to live in a medium contaminated with copper nitrate in laboratory conditions, resisting up to 1000 mg·L^−1^ of copper nitrate in the environment through biosorption. Whether bioremediation is best accomplished via adsorption or biosorption is a matter of debate. Based on our results, we are confident in advocating the use of biosorption as the most efficient method of protection. Although we envision little improvement through adsorption, natural selection has afforded different organisms with increasing adaptability and corresponding capability to manage the intake and removal of inorganic compounds. 

Furthermore, contaminants can accumulate in organisms in two ways. Bioaccumulation occurs when chemicals in the environment are taken up and retained by organisms, mainly through predation [27]. Although the substance can be absorbed orally through consumption of contaminated food, it can also be absorbed through the skin or respiratory system [28]. It should also be noted that contamination by inorganic contaminants can occur through direct absorption from the environment or indirectly through the consumption of contaminated substrate [28]. Biomagnification, on the other hand, occurs through the accumulation of contaminants at different trophic levels by transfer of the contaminant along the food chain until predation of one organism by another accumulates in its body the inorganic contaminant previously absorbed by its prey [27].

Fungi can survive, even with the burden of high concentrations of inorganic pollutants for which they have high absorption tolerance [29]. This ability is maintained by structures that are external or internal to the fungus. Microbial resistance to heavy metals occurs through the accumulation of heavy metals, such as lead, cobalt, and copper, in the vacuoles to form metal carriers [15].

Jakovljević et al. [30] studied the relationship between microorganisms and their ability to form biofilms to biosorb zinc, lead, cadmium, copper, and nickel. Inès et al. [31] demonstrated the potential use of newly identified lipopeptides produced by Bacillus mojavensis BI2 for the bioremediation of heavy metals in contaminated water. *Penicillium chrysogenum* employs multiple stress mechanisms to withstand the effects of both copper and salinity [32]. The Gram-negative bacterium *Cupriavidus basilensis* SRS, which has predatory tendencies, presents mechanisms of resistance to antibiotics, as well as the presence of copper in culture medium [33]. Lacerda et al. [34] studied the absorption capacity of the dead biomass of *Penicillium ochrochloron* and found an ability to act as a biosorbent to remove copper from an aqueous solution. They concluded that the dead biomass of *P. ochrochloron* could be successfully used in the bioremediation of copper in an aquatic environment [34].

Regular consumption of kefir is known to have health benefits. Kefir microorganisms absorb inorganic impurities and excrete them with feces. As a component of kefir, *Penicillium* sp. can absorb inorganic impurities, such as copper, after ingestion and then excrete them through defecation. For this reason, consuming kefir will only have a positive effect on health if taken regularly, as it continuously absorbs the inorganic pollutants that are excreted with kefir in the bowel movement [12].

A predictive toxicological analysis shows that humans resist up to 25 mg·kg^−1^ of copper in the environment (Table 3), and in this work, it was found that *Penicillium* sp. is completely inhibited at a concentration of 1000 mg·L^−1^ of copper under laboratory conditions. These data show that the consumption of kefir, which contains the copper-absorbing microorganism *Penicillium* sp., can protect against overexposure to copper-contaminated food by 100%, even though humans can only tolerate 25 mg·kg^−1^ of copper in the environment.

In a stirred liquid medium that supports the growth of *Penicillium* sp., the addition of 400 mg·L^−1^ copper nitrate dropped the pH from 7.0 to 4.33. When the concentration increased from 400 to 800 mg·L^−1^ Cu(NO_3_)_2_, the pH dropped again to 3.37, as shown by the reaction of adherent cells in Figure 1. While the pH in liquid media did drop to 3.37 with stirring (Table 4), no significant differences were observed between treatments and different pH values in solid media (Table 2). These results strongly suggest that water pollution by inorganic contaminants causes negative effects from the first levels of the food chain to the top of this chain through biomagnification [28]. Thus, the consumption of fish from contaminated environments also poses risks to human health [35]. However, kefir consumption could again play a protective role because the bioremediation, or biosorption, of water-borne pollutants also occurs in microorganisms such as *Penicillium* sp., not the human body. This provides protection for populations that suffer from exposure to contaminated water sources or ingestion of copper accumulated in fish through biomagnification.

Environmental factors, such as pH, temperature, and ionic strength, can impact the effectiveness of bioremediation [24]. According to Lau et al. [36], temperature is among the factors that affect the biosorption of metals, but its effects are less impactful than those when pH changes. The absorption of inorganic contaminants is pH-dependent by affecting the structural chemistry of substances from the culture media and affecting the acidic nature of functional groups on the cell surface [21]. Therefore, pH changes the charge of surface groups on the cell surface, affecting the efficiency of biosorption of heavy metal ions [37,38,39]. More specifically, as pH increases, these functional sites are deprotonated [40]; thus, their negative charge increases, which facilitates binding to cations. In contrast, when a decrease in pH occurs, positive charges increase on the cell surface, and the attraction between biomass and metal ion is reduced. Therefore, the optimal pH for growth in solid media is 4.0, but activity and sporulation occur at pH 10 and pH 9, respectively. In a recent study, it was observed that copper biosorption by *Spirogyra* sp. increased from 31% to 86% when pH increased from 1.0 to 7.0 [41]. In our experiment, an increase in activity occurred at pH 4.0 (Table 2). This occurred both in radial growth and in a liquid medium with agitation. Therefore, contrary to previous consensus, a decrease in the pH of the medium caused better growth (Table 2).

Through SEM, Martinelli and Santos [42] analyzed the morphological structures relevant for the identification of the main nematode fungi, while Juříková et al. [43] used SEM to monitor fungal hyphal cells and subsequently map molecular biomarkers. In the present study, SEM analysis showed the effect of increasing copper concentration on the culture medium in *Penicillium* sp. by the decrease in sporulation compared to that observed in the culture medium in the absence of copper nitrate (Figure 1a). More specifically, SEM was used on biomass samples cultured with shaking for 120 h. In stirred media, the pH was lowered from 7.0 to 4.33 using 800 mg·L^−1^ of copper nitrate. Thus, SEM shows how increasing the concentration of Cu(NO_3_)_2_, affects the biomass of *Penicillium* sp. through increasing bioactivity. This provides morphological evidence that biomass can act as an important environmental cleanser of toxic heavy metal ions, making the use of unsafe chemical processes unnecessary. Thus, bacterial agents were identified that can serve as potential in situ bioremediation agents [35].

## 5. Conclusions

While copper plays an important role, for example, in the construction of myelin, its excess in the body can cause neurological disorders and even Alzheimer’s disease. However, in the present work, we have demonstrated that the use of *Penicillium* sp. for bioremediation under laboratory conditions could counteract the harmful effects of copper. This study lays the groundwork for the selection of candidate microorganisms for bioremediation of copper-contaminated environments. We concluded that *Penicillium* sp. isolated from kefir grains could successfully perform bioremediation through biosorption under laboratory conditions, and we suggested that consumption of the microorganism through kefir milk could similarly minimize the harmful effects of ingesting heavy metal intake because *Penicillium* sp. directly absorbs copper from the medium.

## Figures and Tables

**Table 1:** Growth of *Penicillium* sp. isolated from kefir grains (Pkg) in malt-agar medium (2%) with different concentrations of Cu(NO_3_)_2_.

	CTR mg/L^−1^	400 mg/L ^ns^		500 g/L *		600 mg/L ***		800 mg/L ***		ANOVA
*Penicillium* sp.	D (mm)	D (mm)	IC (%)	D (mm)	IC (%)	D (mm)	IC (%)	D (mm)	IC (%)	F
	90 ^a^	73 ± 1.67 ^a^	19	51.50 ^b^ ± 0.50	43	40.33 ^c^ ± 1.89	55	22.17 ^d^ ± 1.75	75	8.57 ***

D = diameter in millimeters; % IC = inhibitory concentrations percent; ns = no significance compared to CTR (control); * = *p* > 0.05; *** = *p* > 0.001; ^a, b, c, d^ = differences among treatments.

**Table 2:** Growth in millimeters (mm) of *Penicillium* sp. in 2% malt-agar medium with 800 mg·L^−1^ of copper nitrate at pH 4.0, 7.0, and 9.0.

Treatment	48 h	IC%	72 h	IC%	96 h	IC%	120 h	IC%	F
CTR	20.00 ± 1.26		45.00 ± 6.33		18.00 ± 1.28		90.00		932.85 **
Cu pH 4.0	07.25 ± 1.18	6	12.00 ± 0.58	6	14.00 ± 1.76	5	24.40 ± 0.5	73	
Cu pH 7.0	6.00 ± 1.06	28	13.00 ± 1.0	3	19.00 ± 1.83	2	22.50 ± 1.9	75	
Cu pH 9.0	5.00 ± 0.65	24	14.00 ± 20.10	19	15.00 ± 1.16	18	20.66 ± 0.76	77	

CTR = control (*Penicillium* sp. in 2% malt-agar medium with 0 mg Cu(NO_3_)_2_) and *Penicillium* sp. in 2% malt-agar medium with 800 mg Cu(NO_3_)_2_; ** = *p* > 0.05.

**Table 3:** Toxicological prediction of Cu(NO_3_)_2_.

Toxicological Prediction	
Toxicological class	2
LC_50_	25 mg·kg^−1^
Molecular weight	187.56
Number of hydrogen acceptors	6
Number of atoms	9
Number of connections	6
Molecular polar surface area (PSA)	137.76

**Table 4:** Biomass *of Penicillium* sp. grown in liquid media with 0 to 800 mg·L^−1^ of Cu(NO_3_)_2_.

Treatment	Cu(NO_3_)_2_	Biomass (µg)	Inhibition (%)	pH
1	(control group)	578 ± 2.75		7.00
2	Cu^2+^ 400 mg	257 ± 3.00	56%	4.33 ± 0.032
3	Cu^2+^ 500 mg	156.67 ± 2.52	73%	4.19 ± 0.025
4	Cu^2+^ 600 mg	55.33 ± 0.58	90%	4.08 ± 0.003
5	Cu^2+^ 800 mg	41.67 ± 2.08	93%	3.37 ± 0.078

## Data Availability

The datasets used and/or analysed during the current study are available from the corresponding author on reasonable request.
